# Automatic diagnosis of heating in oil-filled terminals of cables

**DOI:** 10.1038/s41598-025-22506-0

**Published:** 2025-11-05

**Authors:** Shunyu Yao, Zhen Liu, Yong Zhao, Shuo Ni

**Affiliations:** 1 State Grid Henan Electric Power Company, Zhengzhou Power Supply Company, Zhengzhou, 450000 China; 2 School of Electrical and Electronic Engineering, North China Electric Power University, Beijing, 102206 China; 3 Department of Mechanical Engineering, Inha University, Incheon, 22212 South Korea

**Keywords:** Cable terminal, Infrared image, Visible light image, Object detection, Heating area, Electrical and electronic engineering, Computer science

## Abstract

A new automatic diagnosis technique was proposed to address the problem of cable oil-filled terminal heating. The infrared and visible light images collected by DJI drones were registered using the scale-invariant feature transform (SIFT) algorithm. The progressive infrared and visible image fusion network based on the illumination aware (PIA Fusion) network was used to fuse the infrared and visible light images. The fused images were used to train the version 5 of You Only Look Once (YOLOv5) network for object detection. The areas prone to heating were identified and mapped to the infrared images before fusion, and the mapped areas of the infrared images were cropped. The cropped images were fed into the DJI infrared analysis software toolkit Thermal Software Development Kit (TSDK) to obtain the temperature information, and diagnosis was performed according to the relevant standards. The infrared images and fused images were separately used for training. The experimental results showed that the mean average precision calculated when the intersection over union (IoU) threshold was 0.5 (mAP@0.5) was 95.3% when training with fused images and the average detection time was 12 ms per image. This technique could replace traditional manual diagnosis to improve detection efficiency and precision.

## Introduction

Compared to overhead lines, cable lines offer many advantages such as the low land occupation rate, resulting in their wide adoption in transmission networks^1–12^. Cable terminals are devices installed at the ends of cables to connect cables to other electrical equipment^13^. Compared to the cable itself and intermediate joints of cable lines, cable terminals suffer from higher failure rates. According to the failure statistics in a provincial capital region in central China, since 2019, the number of terminal failures has accounted for more than 80% of all non-external failures. In recent years, the issue of cable terminal defect diagnosis has attracted significant attention from researchers and maintenance operators. Currently, transmission cable lines mostly use oil-filled terminals. The structures of oil-filled terminals are rather complex, with multiple interfaces of insulating materials and metal connection parts. Defects inside an insulating material or between different insulating materials, or poor contact between the metal connecting parts, can lead to the generation of heat. In routine maintenance, infrared thermal imaging technology is adopted to obtain infrared images of the terminals, which are manually checked to diagnose heating defects. In compliance with the technical specification published in^14^, 110 kV and 220 kV cable lines must be inspected once within 1 month after commissioning, and then every 6 and 3 months, respectively. With the growing number of cable lines, the manual diagnosis of cable terminal heating defects poses issues such as low efficiency and uneven defect detection rate relying on the experience of maintenance personnel. If a cable terminal heating defect is not diagnosed in time, it may develop further into insulation breakdown, leading to the outage of the cable line, a reduction in power supply reliability, a negative influence on society, and huge economic losses^15^. Therefore, it is necessary to study the automatic diagnosis technology of cable oil-filled terminal heating defects, to replace manual diagnosis, improve the defect diagnosis rate, and shorten the diagnosis time.

Several studies have assessed the technology of automatic diagnosis of heating defects in electrical equipment. In^16^, the Canny algorithm was used to identify cable terminals in infrared images, the k-means algorithm was used to extract the suspected overheated areas, based on which templates were constructed, and then suspected overheated areas were matched with the reference phases in the infrared images by the template matching method, to provide interphase temperature comparison for diagnosis. In^17^, the faster region-based convolutional neural network (RCNN) algorithm was used to identify cable terminals in infrared images, and the mean-shift algorithm was used to extract overheating areas. In^18^, the pyramid feature attention network was added to the top-down sampling process of the original pyramid shaped YOLOv3 network to identify insulators in infrared images. In^19^, an improved YOLOv3 target detection method was proposed to identify and locate four types of electrical equipment as well as their abnormal heating areas, and the presence of defects in equipment were diagnosed by calculating the overlap between the identified equipment areas and abnormal heating areas. The above methods used infrared images alone to train the target recognition network to identify electrical equipment. In^16,17^, clustering algorithms were used to extract the overheating area for diagnosis. In^19^, abnormal heating areas were also identified with the object detection algorithm.

However, these methods present three problems. First, visible light and infrared images have their own advantages and disadvantages. The former can provide visual details, but may lose key information, as it is sensitive to factors such as weather. The latter has advantages in anti-interference and night imaging, but typically has low resolution and serious distortion in areas with rich textures. Thus, it is difficult to achieve good recognition results using only infrared images for object detection^20–25^. Second, the method of directly extracting overheated areas cannot accurately diagnose heating defects in cable terminals. An oil-filled cable terminal consists of different materials, such as metal, solid, and liquid insulation, in which the heating defects can cause different temperature increases. Taking stress cones as an example, if a defect in a stress cone leads to higher heat generation, the temperature increase on the terminal surface may be quite small because the heat must be conducted through the stress cone cover, insulating oil, and terminal sleeve. The temperature increase of the stress cone may be ignored when extracting overheated areas, resulting in the missed detection of heating defects. Third, the number of infrared images of equipment with abnormal heating areas is much less than that of those without abnormal heating areas, making it difficult for the model to fully learn and accurately identify the abnormal heating areas.

To address the above issues, automatic diagnosis technology for heating defects in cable oil-filled terminals based on a progressive infrared and visible image fusion network based on illumination aware (PIA Fusion) and the version 5 of You Only Look Once (YOLOv5) was proposed in this work. First, each infrared image and visible light image was registered using the scale-invariant feature transform (SIFT) algorithm to obtain a pair of infrared and visible light images of the same size and with matching pixels. Then, the infrared image and visible light image were fused using the PIA Fusion algorithm. The fused images were preprocessed to produce a fused data set. LabelImg was used to annotate the four heat-prone areas of a cable terminal, namely, the outlet fitting, stress cone, tail pipe, and grounding box. Finally, YOLOv5 was used for training to obtain the corresponding YOLOv5 detection model. In actual applications, the fused images were input into the YOLOv5 detection model for target recognition to identify the heat-prone areas. Then the target areas were mapped to the infrared images without fusion, and then the images were cropped. The cropped image data were input into the Thermal Software Development Kit (TSDK) to obtain the temperature information of the target areas, and finally, defect diagnosis was performed according to the relevant industry standards.

### Heat-prone areas

**Fig. 1:**
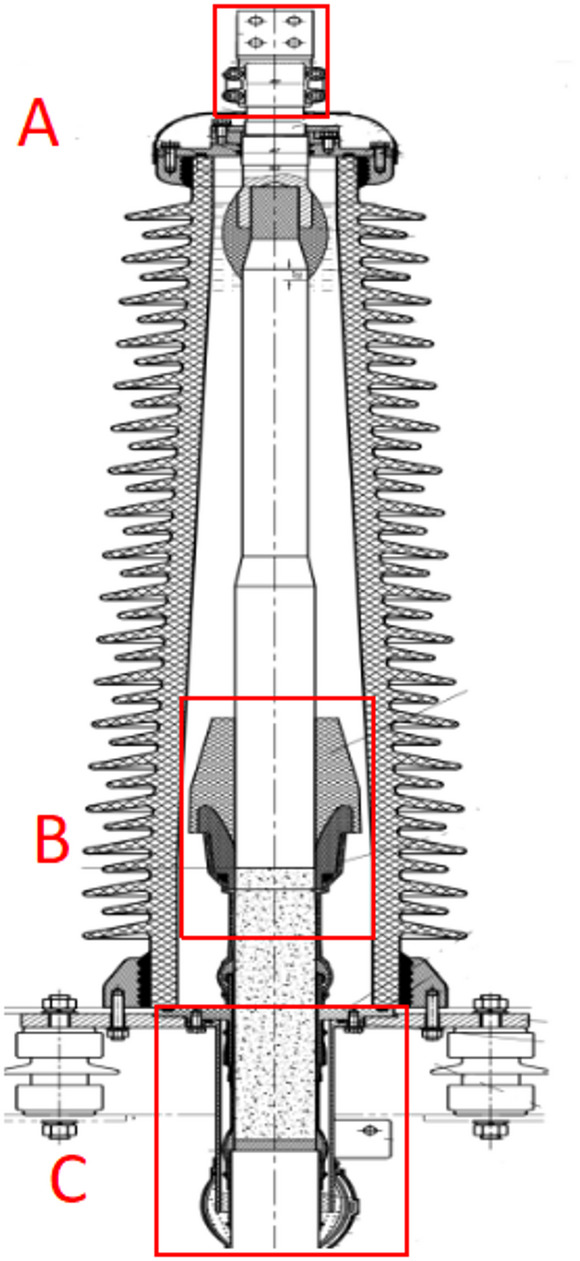
Structural diagram of a oil-filled terminal.

There are two kinds of oil-filled terminals of Cables, namely the Japanese-style terminal and the European-style terminal. The former has a stress cone cover and a cone bracket, while the latter does not, and the other structures of them are similar. As an example, the structure of a European-style terminal is shown in Fig. 1, where three heat-prone areas are labled as A, B and C. In addition, the grounding box is also one of the heat-prone areas.

The outlet fitting, used for leading out the cable core, was labeled as Heating Area A. Heating Area C was the metal tailpipe, which was connected to the metal sheath of cable through lead sealing. The metal tailpipe was also equipped with a terminal for connecting the grounding cable. Additionally, grounding boxes contained multiple bolt connections. Those three heat-prone areas all contained metal connection parts which were prone to heating under the condition of poor connection during operation.

Heating Area B was the stress cone section, which was a non-metallic component. The stress cone was installed at the end of cable insulation shield to control the excessively concentrated electric field intensity of the end. If the stress cone failed to control the electric field intensity, this area was susceptible to heating.

## Methods

### Image registration based on SIFT

Current infrared thermal imaging equipment on the market can simultaneously obtain infrared and visible light images. However, the resolution of infrared images is generally lower than that of visible light images, and the field of view of the visible light images is larger than that of the infrared images. The thermal imaging version of the Mavic3T drone used in this work was used as an example. The size of the captured infrared images was 640 × 512, while the size of the captured visible light images was 4000 × 3000. In addition, because capturing the infrared and visible light images could not be completely synchronized, deviation was present in the visible area between the infrared image and visible light image of the same image pair. PIA Fusion allowed for only the fusion of infrared and visible light images of the same size and matched pixels, therefore, image registration was required first. In this study, each pair of infrared and visible light images was registered based on the SIFT algorithm.

SIFT extracted the position, scale, and rotation-invariant features in different images, mainly through four steps: detection of extrema in scale space, location of keypoints, determination of main orientation of keypoints, and construction of keypoint descriptors^26^.

**Fig. 2:**
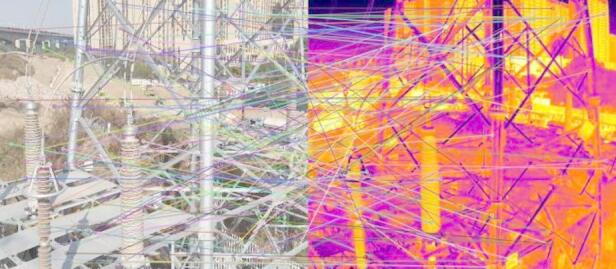
A pair of infrared and visible light images after SIFT matching.

2000 keypoints were chosen for each image, and a 128-dimensional feature vector was formed for each keypoint. For each pair of images, it was judged to be successfully aligned when the aligned keypoints in the visible light and infrared image of the pair were greater than 500. The visible images in the successfully aligned pairs were chopped according to the smallest outer rectangle of all the aligned keypoints, and then the cropped visible images were scaled to 640 × 512. As a result, pairs of infrared and visible light images with the same size and pixel matching were obtained. A pair of infrared and visible light images was registered and cropped, after which the matching keypoints were connected, as shown in Fig. 2.

### Image fusion based on PIA fusion

After a pair of infrared and visible light images was registered through the SIFT algorithm, the results would not be satisfactory if the corresponding pixels of the two images were simply added together for fusion. PIA Fusion could effectively maintain the brightness distribution of the target and the texture information of the background^27^, resulting in better fusion results, and creating a good foundation for subsequent object detection.

**Fig. 3:**
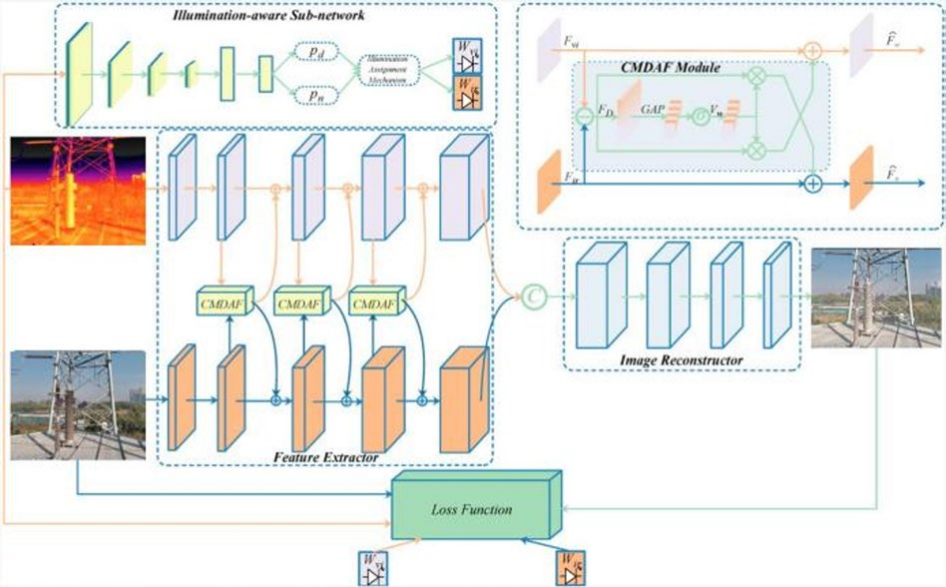
PIA Fusion network framework^27^.

As shown in Fig. 3, the network framework of PIA Fusion mainly included a feature extractor and image reconstructor. The feature extractor was used to extract features from infrared and visible light images, respectively, and progressively fuse them. The fused features were used to generate a fused image through the image reconstructor. The feature extractor contained an illumination-aware subnetwork.

**Fig. 4:**
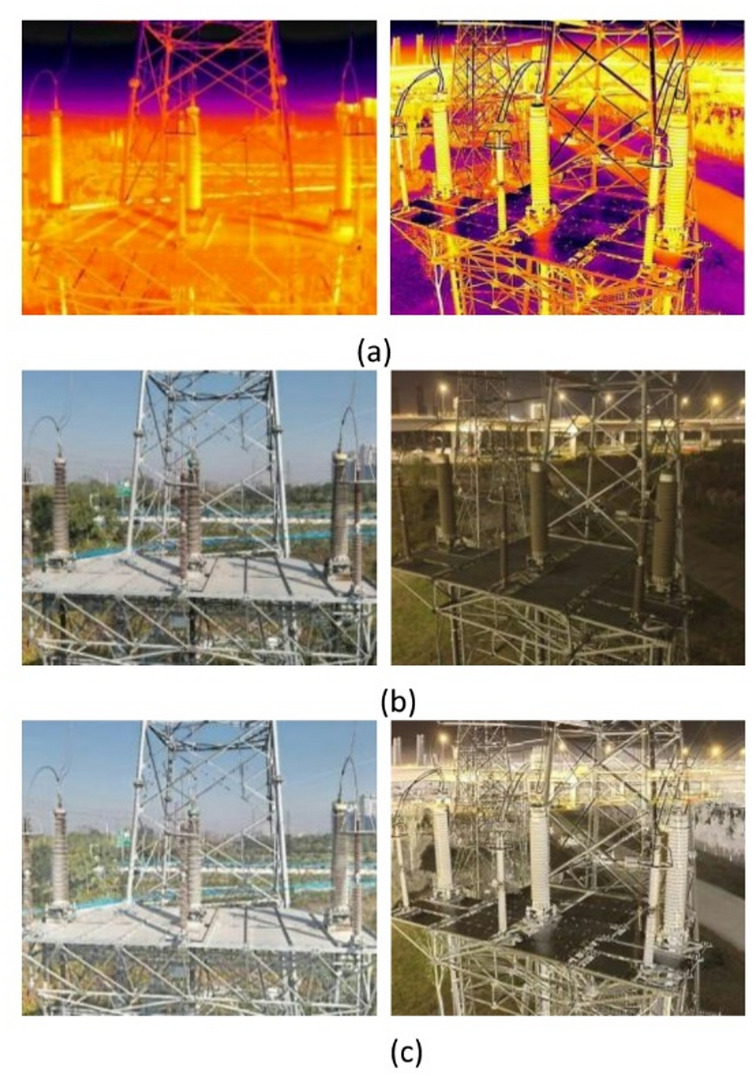
Two pairs of infrared and visible light images fused by PIA Fusion: (**a**) infrared image; (**b**) visible light image; (**c**) fused image.

Two pairs of infrared and visible light images were registered and cropped, and then fused by PIA Fusion, as shown in Fig. 4. The pair on the left and the pair on the right consisted of the infrared, visible light, and fused images captured in the daytime and nighttime, respectively.

### Object detection based on YOlOv5

YOLOv5 consists of a single-stage object detection algorithm. While retaining the speed advantage of the YOLO series algorithms, the precision was significantly improved compared to the previous versions. Multiple versions of the YOLOv5 algorithm exist, and this work was based on the YOLOv5 6.0 version. YOLOv5 6.0 version contained five network models of different sizes: YOLOv5n, YOLOv5s, YOLOv5m, YOLOv5l, and YOLOv5x. YOLOv5s was used in this study for model training and object detection.

**Fig. 5:**
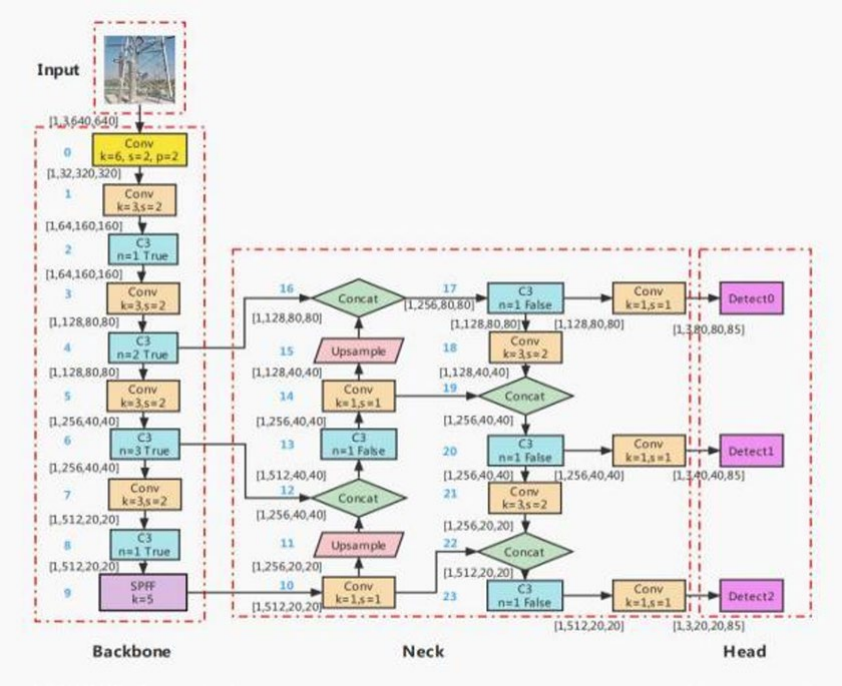
Main architecture of the YOLOv5 6.0 model.

As shown in Fig. 5, the YOLOv5 6.0 model mainly consisted of four components: Input, Backbone, Neck, and Head.

The Input was the image input module, while the Backbone was mainly used to extract features from an image and form a feature map, which was then integrated by the Neck. The Head used three feature maps of different scales output by the Neck to predict the type, confidence, and detection box position of the targets for large, medium, and small targets, respectively.

The Input of YOLOv5 used Mosaic enhancement to improve the model’s ability to detect small objects. The prior anchor box calculation function was embedded in the entire training code. Before each training started, the prior anchor box calculation was adaptively performed according to the different data sets. The cross stage partial network (CSPNet) was used in the Backbone and the Neck to enhance the model’s learning ability and reduce memory costs and eliminate computing bottlenecks. The Neck adopted the structures of feature pyramid networks (FPN) and pyramid attention networks (PAN) to enhance the fusion of graphic features and semantic features.

**Fig. 6:**
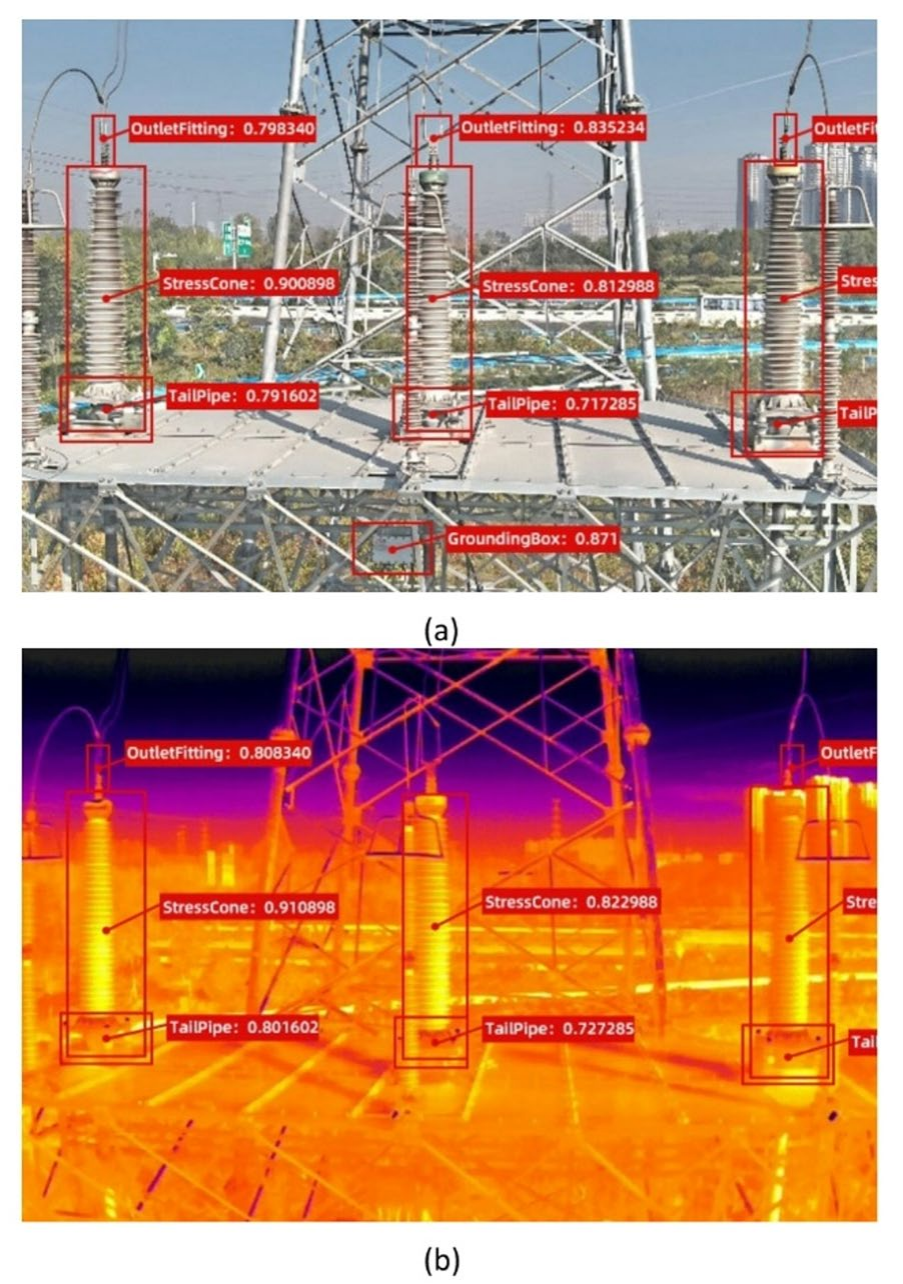
Detection result using YOLOv5: (**a**) fused image; (**b**) infrared image.

The fused image was detected by YOLOv5. Four areas prone to heat generation were identified, namely, the outlet fittings, the stress cones, the tail pipes, and the grounding box, as shown in Fig. 6a. Figure 6b presents the result of the infrared image detected by YOLOv5. Compared to Fig. 6a, the grounding box and outlet fitting of the middle phase terminal remained undetected in Fig. 6b. Aerial inspection of the cables is generally conducted in daytime, due to poor line of sight at night, posing a certain risk of accidentally touching live equipment and causing failure. Therefore, all images used in this experiment were taken during the day. What is more, the stress cones detected here were actually the whole sleeves of the cable terminals, lager than the actual stress cone areas. Further detection was conducted to identify every skirt of the sleeves, and the actual stress cone areas were located according to the skirts of the sleeves.

### Temperature data extraction and defect diagnosis

**Fig. 7:**
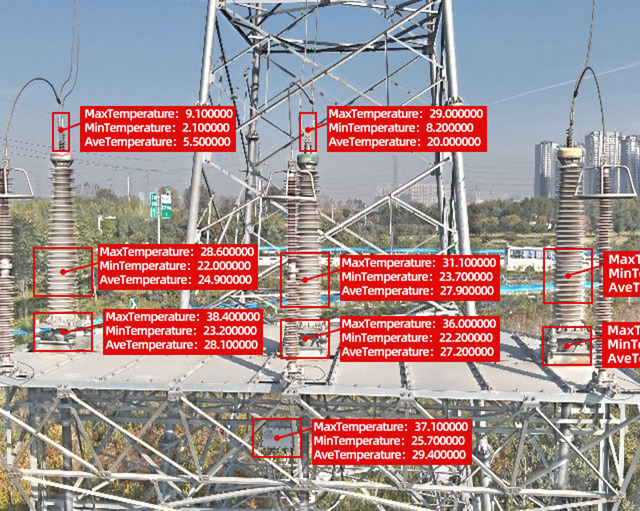
Detection result with temperature information annotated.

As shown in Fig. 7, from the detection results obtained by YOLOv5, using the output location information of each target area, the corresponding area in the infrared image before fusion was identified and cropped. The cropped image was then input into the DJI UAV infrared thermal imaging temperature extraction program TSDK, which automatically output the highest temperature, lowest temperature, and average temperature of the area. For example, in the detection results in Fig. 6, the temperature information was labeled on the detection result map shown in Fig. 7. The highest temperature in each area was used for interphase comparison or comparison with the threshold.

According to^14^, the infrared temperature measurement diagnosis criteria could be divided into one for metal connection parts and one for terminals and joints. For the metal connection parts, an interphase temperature difference greater than or equal to 10 °C was regarded as defective, between 6 °C and 10 °C was regarded as abnormal, and less than 6 °C was regarded as normal. For terminals and joints, an interphase temperature difference greater than or equal to 4 °C was regarded as defective, between 2 °C and 4 °C was regarded as an abnormal value, and less than 2 °C was regarded as a normal value. For the outlet fittings and tail pipes, interphase comparison was performed using the same criteria as the metal connection points. For the stress cone parts, interphase comparison was performed according to the standards of terminals and joints. For the grounding boxes, the highest temperature was compared with the background temperature for diagnosis. A temperature difference greater than 10 °C was regarded as defective, between 5 °C and 10 °C was regarded as an abnormal value, and less than 5 °C was regarded as a normal value, according to^28^.

## Analysis and evaluation of experimental results

### Data collection and preprocessing

The data used in the experiment were collected from nearly 200 towers of 110 or 220 kV oil-filled cable terminals in a region in central China. A DJI thermal imaging version of the Mavic3T drone was used. The drone was equipped with a telephoto visible light camera, a short-focus visible light camera and an infrared thermal imaging camera, which were all towards the same direction. The infrared images were taken by the infrared thermal imaging camera, and the visible light images were taken by the short-focus visible light camera, which was at the upper right corner of the infrared thermal imaging camera, and the distance between the centers of the two cameras was about 1.5 to 2 cm. The infrared thermal imaging camera automatically generated images in JPEG format. The targets for data collection consisted of oil-filled cable terminals and grounding boxes.

During the data collection process, the drone acquired a total of 7052 pairs of visible light images and infrared images. Each pair contained a visible light image and an infrared image taken simultaneously. The collected images were processed with noise reduction to improve the image quality, and 6972 pairs of images were screened out by judging whether the mean square error and peak signal-to-noise ratio of the noise reduced images met the standard. After the SIFT algorithm was used to complete the registration of the 6972 pairs of images, the images were fused using PIA Fusion to obtain 6972 infrared and visible light fusion images. Then the infrared images and fusion images were annotated separately. The outlet fittings, stress cones, tail pipes, and grounding boxes in each image were annotated separately. Finally, two sets of labeled images are obtained, each with 6972 images. The ratio of the training set, validation set, and test set was set to 4:1:1.

### Experimental parameters and evaluation metrics

The experiment was run on a computer with a 64-bit Ubuntu 20.04 operating system, equipped with an RTX-4070 graphics card with 8 GB of video memory, an Intel i9-13980HX processor, and 16 GB of running memory. Training was performed on the PyTorch framework. The YOLOv5s network model was used to obtain the single-backbone network pre-training model parameters. The infrared image training set and fusion image training set were used for training, respectively. All input images had a size of 640 × 640, and the batch size was 4. The initial learning rate was 0.01, the decay rate was 0.0005, and the training process was accelerated by GPU.

For the experimental results, the average time taken to identify a single image in the same test set was used to measure the detection speed, and the average precision (AP) of a single class label and the mean average precision (mAP) of all class labels were used to evaluate detection precision. In addition, the change curve of network loss was plotted to present the trend of network loss changes with the number of iterations.

### Analysis of the experimental results

The detection precision results are shown in Table 1. Compared to applying infrared images for training and detection, the average precision of the four types of labels, namely, outlet fitting, stress cone, tail pipe and grounding box, and the mean average precision of all class labels were improved when using the fused images. Specifically, the average precision for the grounding boxes and the outlet fittings improved the most, by 19.5% and 11.8%, respectively, while the mean average precision of all class labels increased by 8.6%.

**Table 1:** Comparison of detection Precision.

Title 1	Infrared images	Fused images
AP ^1^for outlet fittings	86.2%	98.0%
AP for stress cones	94.9%	96.4%
AP for tail pipes	96.2%	97.8%
AP for the grounding boxes	69.4%	88.9%
mAP@0.5 ^2^	86.7%	95.3%

^1^AP denotes the average precision of a single class label.

^2^mAP@0.5 denotes the mean average precision calculated when the IoU (Intersection over Union) threshold is 0.5.

In terms of detection speed, the average recognition time for a single infrared image and a single fused image was 13 and 12 ms, respectively. Applying fused images for detection was slightly faster than the infrared images.

**Fig. 8:**
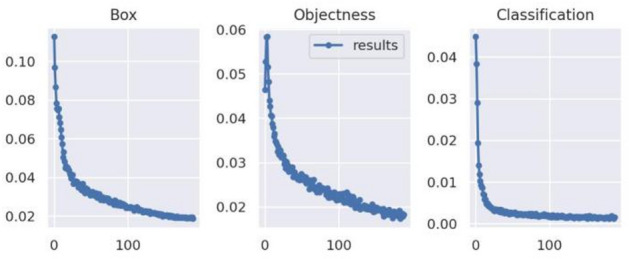
Change curve of network loss for training using infrared images.

**Fig. 9:**
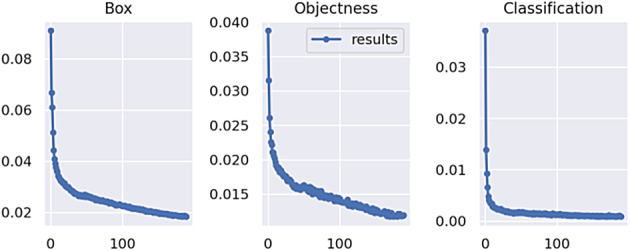
Change curve of network loss for training using fused images.

The change curves of network loss during training are shown in Figs. 8 and 9. Compared to training with infrared images, training with fused images led to a faster decrease and smaller data fluctuations of bonding box loss, confidence loss, and classification loss of the network.

In summary, compared to applying infrared images, using fused images for training and detection slightly improved the recognition speed while significantly increasing the rate of decrease of network loss during training. More importantly, detection precision was greatly improved, with mAP@0.5 reaching 95.3%.

## Conclusions

- Based on existing automatic diagnosis methods applying infrared images to conduct objection detection and overheating area extraction, in this work, we proposed an automatic diagnosis technology based on PIA Fusion and YOLOv5 for the heating defects of cable oil-filled terminals. The steps included infrared and visible light image registration, image fusion, object detection, temperature extraction of areas prone to heating, and defect diagnosis.
- Fused images were used for training and detection. Compared to infrared images, although the detection speed slightly improved, mAP@0.5 improved by 8.6%.
- The four heat-prone areas of the cable oil-filled terminal, namely, the outlet fittings, stress cone, tail pipe, and grounding box were detected, and the existing diagnostic standards were more accurately benchmarked to achieve accurate diagnosis.
- The technology proposed in this study could reduce the dependence of cable oil-filled terminal heating defect diagnosis on manual labor and human experience, to achieve intelligent automatic diagnosis, improve the work efficiency and defect detection rate, and enhance the lean operation and inspection of transmission cables. This work may benefit the development of intelligent diagnosis of heating defects of electrical equipment.

## Data Availability

The datasets used and/or analysed during the current study available from the corresponding author on reasonable request.
